# Effect of Nasal Inflammation on the Mind

**Published:** 1889-03

**Authors:** Thomas F. Rumbold

**Affiliations:** St. Louis, Mo.


### EFFECT OF NASAL INFLAMMATION ON THE MIND.

BY THOMAS F. RUMBOLD, M. D., ST. LOUIS, MO.

A normal condition of the mind depends upon a normal supply of blood to the normal brain. An abnormal supply of blood, even to the healthy brain, will produce an abnormal condition of the mental faculties. I have noticed for many years that every patient who had suffered a severe attack of cold in his nasal passages, had his olfaction almost completely obtunded; also had his mental powers more or less lowered in their capacity.

Knowing that the blood supply to the superior portion of the nasal passages is received alone from within the brain cavity, and knowing that the peripheral portions of these arteries must be affected injuriously by the inflammation at the same time, if not before, the same central or brain portions are affected, it was not difficult to account for the mental capacity that usually follows cold in the head. It follows that if an acute inflammation in the nasal passages was accompanied by mental manifestations, that mental incapacity of a more severe character must accompany chronic rhinitis. Clinical observation has shown that this is the case. The fact that the brain portions of these arteries take on an abnormal condition at the same time, if not before the nasal portions do, indicates plainly that nasal inflammation and mind trouble must accompany each other.

For quite a number of years the ophthalmologist has described the condition of the brain from observations made with the ophthalmoscope to the peripheral ends of the very same blood vessels that are spread over the superior portions of the nasal passages, the ophthalmic artery. I am very certain that I could make the same diagnosis from a rhinal examination, long before the eyes would give any symptoms that would suggest an ophthalmic examination. I have for many years seen the rhinal vessels decrease in size, under treatment for chronic instillation, and with this decrease, a marked improvement in the eye affection and in the mental ailment, showing plainly that reduction of the rhinal inflammation assisted in the reduction of the abnormal blood supply to the brain.

Cases of this kind, of a marked character, have occurred in my practice, and in my son's practice, almost weekly. As I had been observing this relation between the nasal passages and the brain since 1862, but more especially since 1866, I have asked a large number of physicians in the Eastern cities of this country, and in many of the largest cities in Europe, (in 1881 and 1884) if they had made the same observations, and found that none had their attention drawn to this subject. My impression now is, that not one of the physicians placed any importance in these rhino-mental symptoms or asked any questions concerning them.

In the treatment of common mind troubles, I am sure there is nothing more important to the practitioner, than a knowledge of this relation of the nasal passages to the brain, because there is no other method of treatment, that will as quickly relieve many of the common mind troubles, as the relief of the rhinal inflammation.

---

MRS. VENEERING—"Really, my dear doctor, you must come to my ball. It is Lucy's coming-out affair, you know, and I shall take no refusal; none at all." Doctor Bygfee—"Well, you see, my dear madam, I am a very busy man. My time is not my own—" Mrs. Veneering—"Say no more. Include the visit in your bill. There, I shall expect you. Good-bye."—*Pittsburg Bulletin.*

